# Effects of a Novel Glucokinase Activator, HMS5552, on Glucose Metabolism in a Rat Model of Type 2 Diabetes Mellitus

**DOI:** 10.1155/2017/5812607

**Published:** 2017-01-16

**Authors:** Ping Wang, Huili Liu, Li Chen, Yingli Duan, Qunli Chen, Shoumin Xi

**Affiliations:** ^1^The Key Laboratory of Pharmacology and Medical Molecular Biology, Medical College, Henan University of Science and Technology, Luoyang 471023, China; ^2^School Clinic, Henan University of Science and Technology, Luoyang 471023, China; ^3^Department of Clinical Research & Development, Hua Medicine, Shanghai 201203, China

## Abstract

Glucokinase (GK) plays a critical role in the control of whole-body glucose homeostasis. We investigated the possible effects of a novel glucokinase activator (GKA), HMS5552, to the GK in rats with type 2 diabetes mellitus (T2DM). Male Sprague-Dawley (SD) rats were divided into four groups: control group, diabetic group, low-dose (10 mg/kg) HMS5552-treated diabetic group (HMS-L), and high-dose (30 mg/kg) HMS5552-treated diabetic group (HMS-H). HMS5552 was administered intragastrically to the T2DM rats for one month. The levels of total cholesterol, triglyceride, fasting plasma insulin (FINS), and glucagon (FG) were determined, and an oral glucose tolerance test was performed. The expression patterns of proteins and genes associated with insulin resistance and GK activity were assayed. Compared with diabetic rats, the FINS level was significantly decreased in the HMS5552-treated diabetic rats. HMS5552 treatment significantly lowered the blood glucose levels and improved GK activity and insulin resistance. The immunohistochemistry, western blot, and semiquantitative RT-PCR results further demonstrated the effects of HMS5552 on the liver and pancreas. Our data suggest that the novel GKA, HMS5552, exerts antidiabetic effects on the liver and pancreas by improving GK activity and insulin resistance, which holds promise as a novel drug for the treatment of T2DM patients.

## 1. Introduction

Type 2 diabetes mellitus (T2DM), which accounts for approximately 90–95% of the diagnosed cases of diabetes [1], is a major health problem worldwide. The total number of people with diabetes in 2013 was 382 million, and this number will most likely increase to 592 million by 2035 according to the International Diabetes Federation [2]. T2DM is characterized by elevated fasting plasma glucose (FPG), insulin resistance, increased hepatic glucose production (HGP), and a deficiency in glucose-stimulated insulin secretion (GSIS). The oral therapies that are currently widely used for the treatment of patients with T2DM act mainly by reducing HGP (e.g., biguanides), promoting insulin action (e.g., thiazolidinediones), stimulating insulin release (e.g., sulfonylurea drugs), inhibiting the absorption of intestinal glucose (e.g., *α*-glucosidase inhibitors), and increasing the endogenous glucagon-like peptide 1 (GLP-1) and glucose-dependent insulinotropic peptide (GIP) levels (e.g., sitagliptin and saxagliptin) [3]. These therapies, however, have some notable shortcomings, such as insufficient efficiency, limited tolerability, and significant side-effects [4]. Therefore, effective, safe, and novel treatments that target novel pathways are urgently needed to help patients reach and maintain the optimal plasma glucose levels.

Glucokinase (GK), also known as hexokinase IV or D, is a 50 kDa cytoplasmic enzyme and one of the four hexokinase isozymes that mediates phosphorylation of glucose to glucose-6 phosphate (G-6-P) in the presence of ATP [5]. GK is predominantly expressed in hepatocytes and pancreatic *β*-cells and plays a key role in the regulation of carbohydrate metabolism. Compared with other hexokinases, GK has a lower affinity for glucose (*K*_*m*_ ≈ 8 mmol/L), displays positive cooperativity for this substrate, and is not inhibited by its product G-6-P [6]. GK acts as a “glucose sensor” in *β*-cells for GSIS [7]. In the liver, GK is the rate-limiting enzyme in glucose metabolism and is essential for both glycogen synthesis and glucose production [8]. The genetic mutations of the enzyme in human can cause diabetes or hypoglycemia, which further demonstrates the critical role of GK in glucose metabolism [9].

Nowadays, the development of glucokinase activators (GKAs) as a potential new class of therapeutics for the treatment of T2DM is attractive to researchers. Recently, several GKAs have been discovered, and these GKAs have been shown to effectively improve glucose homeostasis in diabetic animal models and T2DM patients [10–12]. However, clinical studies of GKAs have revealed some undesirable effects, such as hypoglycemia [11], elevated circulating triglyceride (TG) levels [13], and increased blood pressure [11]. Furthermore, it appears that this class of therapeutics does not exhibit a durable response after long-term treatment [11, 14]. In the present study, we investigated the effects of a novel fourth-generation GKA, HMS5552, which has a structurally novel amino-acid-based chemical scaffold, on the improvements of GK activity and insulin resistance in a rat model of T2DM induced by a high-fat diet and streptozotocin (STZ). Although the novel drug is currently in Phase II clinical trials, the results of the current study are not available in the public domain.

## 2. Materials and Methods

### 2.1. Chemicals

HMS5552 (purity > 95% by HPLC) was provided by Hua Medicine (Shanghai, China) and STZ was purchased from Sigma (St. Louis, MO, USA). All other chemicals were of analytical grade.

### 2.2. Animals

Male Sprague-Dawley (SD) rats (aged approximately 6–8 weeks and weighing 200–230 g) were obtained from the Experimental Animal Center of Henan Province (Zhengzhou, China). The rats were housed in an air-conditioned room at 22 ± 2°C (50 ± 10% humidity) and subjected to 12 h light/12 h dark cycles with free access to food and water during the experiments. All of the animals were allowed to adapt to the environment for one week before the experiment and were fed laboratory chow. All protocols conformed to the guidelines from the National Animal Care and Use Committee of China. In addition, all of the animals received human care in compliance with the Principles of Laboratory Animal Care.

### 2.3. Development of High-Fat Diet- (HFD-) Fed and Low-Dose STZ-Treated T2DM Rats

The rats were allocated to one of two dietary regimens: a normal diet or a HFD (68% normal diet, 20% sucrose, 2% cholesterol, and 10% fat). After eight weeks of dietary manipulation, diabetes mellitus was induced in the HFD-fed rats through a single intraperitoneal (i.p.) injection of STZ (40 mg/kg), whereas the control rats were injected i.p. with vehicle (citrate buffer, pH 4.4) in a dose volume of 1 mL/kg. Seven days after the STZ injection, the rats were deprived of food and water overnight, and blood samples were collected from the lateral tail vein. The FPG levels of each rat were measured using an Accu-Chek Active system (Roche Diagnostics, Mannheim, Germany), and the rats with repeated FPG measurements greater than 8.0 mmol/L were considered to have diabetes and selected for subsequent experiments.

### 2.4. Experimental Design

Experimental diabetic rats were successfully established and randomly divided into three groups: diabetic group (*n* = 6), low-dose (10 mg/kg) HMS5552-treated diabetic group (HMS-L, *n* = 6), and high-dose (30 mg/kg) HMS5552-treated diabetic group (HMS-H, *n* = 6). Rats fed a normal diet served as the control group (*n* = 6). HMS5552 dissolved in phosphate-buffered saline (PBS, 100 mmol/L, pH 7.4) was administered intragastrically (i.g.) to the HMS5552-treated diabetic rats daily (8:00 AM) for one month. The diabetic rats and the control rats received equal volumes of PBS and saline, respectively. During the experimental period, the animals in the control group were fed a normal diet, and those in experimental diabetic groups were fed a HFD. The FPG and glucose levels were measured every four days 2 h after HMS5552 administration. An oral glucose tolerance test (OGTT) was performed on day 30, and an oral drug tolerance test (ODTT) was performed on days 1 and 28 in the experimental period. The rats were sacrificed under anesthesia, and samples of blood, liver, and pancreas were immediately collected.

### 2.5. Oral Glucose Tolerance Test (OGTT)

After a 12 h overnight fast, the rats in each group were given glucose at a concentration of 1 g/kg of body weight via gavage. The blood glucose concentrations were determined through the analysis of blood samples collected from the tail vein at 0 (prior to glucose administration), 15, 30, 60, 90, 120, 180, and 240 min after glucose administration.

### 2.6. Oral Drug Tolerance Test (ODTT)

After overnight fasting for 12 h, the FPG levels in each group were measured. HMS5552 at doses of 10 mg/kg and 30 mg/kg was administered i.g. to the rats in the HMS-L and HMS-H groups, whereas the diabetic rats and control rats were treated with PBS and saline, respectively. Blood samples from the tail vein were collected at 30, 60, 120, 180, and 240 min for the measurements of the blood glucose concentrations. After blood sample collection, the rats had free access to food and water, and the glucose concentrations in samples collected from the tail vein at 270, 300, and 360 min (i.e., 30, 60, and 120 min after diet) were determined.

### 2.7. Biochemical Assays

Blood samples were collected in tubes containing 0.1 M ethylenediaminetetraacetic acid (EDTA) as an anticoagulant, and plasma was separated by centrifugation at 3000 ×g for 10 min. The total cholesterol (TC) and triglyceride (TG) levels were determined using commercial diagnostic kits (Mindray, Shenzhen, China). The fasting insulin (FINS) and glucagon (FG) levels were assayed using ELISA kits purchased from Shanghai Elisa Biotech Inc. (Shanghai, China).

### 2.8. GK Activity Assay

GK activity was measured using an enzyme-coupled photometric assay with liver homogenates of different glucose concentrations (0.5, 2.5, 5, 10, 20, 25, 50, and 100 mmol/L) as previously described [15], and correction for the hexokinase activity was applied by subtracting the activity measured at 0.5 mmol/L glucose from the activity measured at 100 mmol/L glucose, and the *K*_*m*_ value was calculated from a fitted curve.

### 2.9. Immunohistochemistry Analysis

Liver and pancreas samples were embedded in paraffin and cut into 4 *μ*m sections. After rehydration, the sections were incubated with 3% H_2_O_2_ for 10 min to block endogenous peroxidase activity. Antigen retrieval was performed in a microwave oven in citrate buffer (10 mmol/L, pH 6), and nonspecific binding sites were then blocked with 5% BSA for 20 min. The liver and pancreas sections were incubated with a rabbit polyclonal antibody against GK (1 : 100 dilution; Bioss, Beijing, China) and a rabbit polyclonal antibody against insulin (1 : 100 dilution; Bioss, Beijing, China), respectively, overnight at 4°C and then treated with a biotinylated secondary antibody (Zhongshan Goldenbridge, Beijing, China) for 30 min. The signal was detected using 3,3′-diaminobenzidine (DAB; Sigma, St. Louis, MO, USA). The sections were then counterstained with hematoxylin and examined microscopically for specific signals, and photographs were taken using a digital image capture system (Olympus, Tokyo, Japan).

### 2.10. Image Analysis for GK and Insulin Immunoreactivity

In order to evaluate GK and insulin immunoreactivity, the GK and insulin-immunopositive cells were counted. A total of 10 random fields were selected in each group, and the total numbers of cells in the hepatocytes, islets of the pancreas, and GK- and insulin-immunopositive cells were counted for each group of rats by Image-Pro Plus 6.0 software (Media Cybernetics, Bethesda, MD). The percentage of GK- and insulin-immunopositive cells was calculated.

### 2.11. Western Blot Analysis

The total proteins from the liver tissues were extracted, and the protein concentrations were determined with a BCA protein assay kit (Pierce Biotechnology, Rockford, IL, USA). Aliquots of the lysates (70 *μ*g of protein) were diluted in sample buffer [50 mmol/L Tris, pH 6.8, 2% sodium dodecyl sulfate (SDS), 10% glycerol, 0.1% bromophenol blue, and 5%  *β*-mercaptoethanol] and boiled for 5 min at 95°C prior to analysis. The samples were then subjected to 12% SDS-polyacrylamide gel electrophoresis (SDS-PAGE) and electrotransferred to PVDF membranes. The membranes were washed three times with PBST (3.2 mmol/L Na_2_HPO_4_, 0.5 mmol/L KH_2_PO_4_, 1.3 mmol/L KCl, 135 mmol/L NaCl, pH 7.4, and 0.05% Tween-20, pH 7.4), blocked with 5% nonfat milk for 30 min at 37°C, and then incubated with a rabbit polyclonal anti-GK antibody (1 : 1000 dilution; Bioss, Beijing, China) overnight at 4°C. After the membranes were incubated with HRP-conjugated secondary antibody at 37°C for 1 h, the protein bands were visualized using DAB and quantified with Gel Pro Analyzer software 4.0 (Media Cybernetics Inc., Bethesda, MD, USA) using *β*-actin as the internal standard.

### 2.12. Semiquantitative RT-PCR

Total RNA from the rat liver was extracted using the TRIzol reagent (Invitrogen, CA, USA) according to the manufacturer's instructions. The purity and concentration of RNA were determined using a NanoDrop ND-1000 spectrophotometer (NanoDrop Technologies, Wilmington, DE, USA). cDNAs were synthesized from 1 *μ*g of total RNA using a PrimeScript 1st strand cDNA Synthesis Kit (TaKaRa, Dalian, China) according to the manufacturer's recommended protocol. PCR was performed with 2x Es Taq MasterMix (CWBIO, Beijing, China) in an MJ Mini Gradient Thermal Cycler (Bio-Rad) using the following parameters: 94°C denaturation for 3 min followed by 35 cycles of 95°C for 30 s, 60°C for 30 s, and 72°C for 30 s and a final extension at 72°C for 3 min. GAPDH was amplified from the same cDNA samples as an internal control. The amplified PCR products were analyzed in 2% agarose gels, and a semiquantitative analysis of the band intensities was performed with Gene Tools software (UVP, Inc., Upland, CA, USA). The intensities of the bands were normalized against that of GAPDH. The following primer sequences for RT-PCR were used: GK F, 5′-TCAACTACAGAAAATGGCGGAA-3′, and R, 5′-CCAGAACTGTAAGCCACTCG-3′; GAPDH F, 5′-ATTCAACGGCACAGTCAA-3′, and R, 5′-CTTCTGGGTGGCAGTGAT-3′.

### 2.13. Statistical Analysis

All of the data are expressed as the mean ± standard deviation (SD). The values obtained before and after treatment within each group were analyzed using paired Student's *t*-test. Comparisons between groups were performed through one-way ANOVA followed by Tukey's post hoc tests. All statistical analyses were performed using SPSS 17.0 (SPSS Inc., Chicago, IL, USA). Values of *P* < 0.05 and *P* < 0.01 were considered statistically significant and highly significant, respectively.

## 3. Results

### 3.1. Effects of HMS5552 on FPG Levels

**Figure 1:**
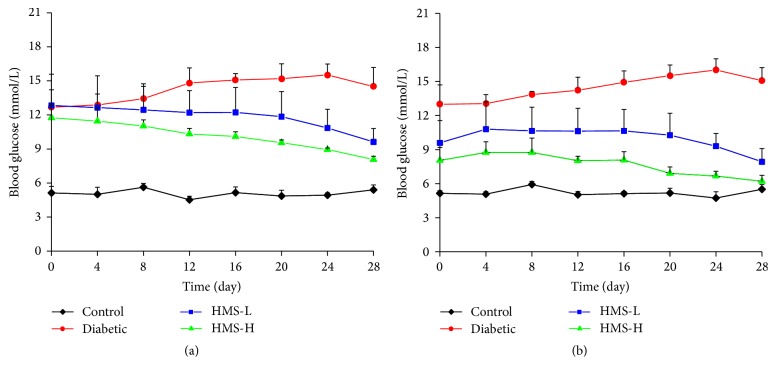
Changes in the fasting blood glucose levels (a) and glucose levels 2 h after HMS5552 administration (b) in the rats of the different groups. HMS-L group, low-dose (10 mg/kg) HMS5552-treated diabetic group; HMS-H group, high-dose (30 mg/kg) HMS5552-treated diabetic group. The data are expressed as the mean ± SD (*n* = 6).

As shown in Figure 1(a), high FPG levels were detected in the rats belonging to all of the experimental groups at the beginning of the experiment (day 0). Two hours after a single i.g. injection of 10 mg/kg or 30 mg/kg HMS5552, the glucose levels of the HMS-L and HMS-H groups were decreased by ~25% and 31%, respectively, compared with the levels detected prior to HMS5552 administration (*P* < 0.05; Figure 1(b)). After 27 d of treatment, significant decreases in the FPG levels were observed in the HMS5552-treated groups, and reductions of ~18% and 23% in the glucose levels were measured 2 h after HMS5552 administration in the HMS-L and HMS-H rats, respectively, compared with the levels detected prior to HMS5552 administration (all *P* < 0.05). These results suggest that HMS5552 exerts a glucose-lowering effect on the glucose levels in diabetic rats.

### 3.2. Effects of HMS5552 on Biochemical Parameters

The effects of HMS5552 on the levels of FINS, FG, TG, and TC in each group of rats were shown in Table 1. As can be seen from the table, the levels of FINS, TG, and TC were significantly higher in the rats belonging to the diabetic group than in those of the control group (all *P* < 0.01). After treatment with 10 mg/kg and 30 mg/kg HMS5552, the levels of FINS were significantly decreased compared with those found in the diabetic group (all *P* < 0.01), whereas the levels of TG and TC were not significantly changed. The FG levels presented the opposite pattern: these were significantly decreased in the diabetic rats compared with the normal controls (*P* < 0.01) and increased in the HMS-L and HMS-H groups compared with the diabetic rats.

### 3.3. Effects of HMS5552 on Oral Glucose Tolerance

**Figure 2:**
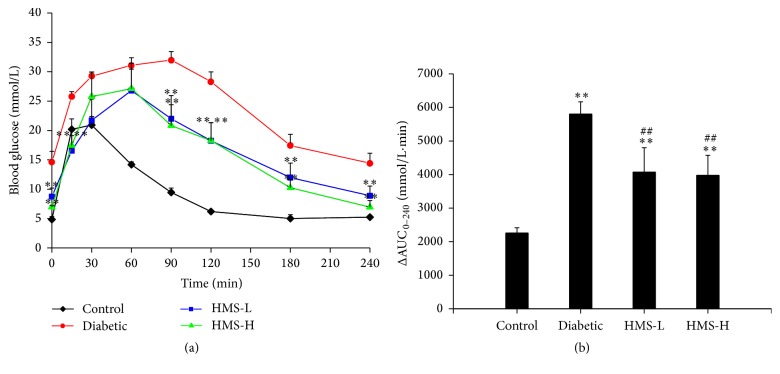
Effects of HMS5552 on the OGTT (a) and the ΔAUC_0–240 min_ obtained from the OGTT (b). HMS-L group, low-dose (10 mg/kg) HMS5552-treated diabetic group; HMS-H group, high-dose (30 mg/kg) HMS5552-treated diabetic group. The data are expressed as the mean ± SD (*n* = 6). ^*∗∗*^*P* < 0.01, compared with the control group; ^##^*P* < 0.01, compared with the diabetic group.

To investigate changes in glucose tolerance among the different groups, an OGTT was performed. As shown in Figure 2(a), the blood glucose levels of the control rats reached a peak at 30 min after the administration of glucose and gradually decreased to the pre-glucose load level at 120 min. In the diabetic group, glucose tolerance was impaired because the FPG level was 3-fold higher than that of the control group. The peak was observed at 90 min, and the baseline glucose value was not restored at 120 min. The HMS-L and HMS-H rats exhibited significant attenuation of serum glucose at 0, 15, 90, 120, 180, and 240 min compared with the diabetic rats (all *P* < 0.01). Furthermore, the areas under the plasma glucose curves from 0 to 240 min (ΔAUC_0–240 min_) obtained for the HMS-L and HMS-H rats were also significantly reduced compared with that of the untreated diabetic rats (all *P* < 0.01; Figure 2(b)).

### 3.4. Effects of HMS5552 on Drug Tolerance

**Figure 3:**
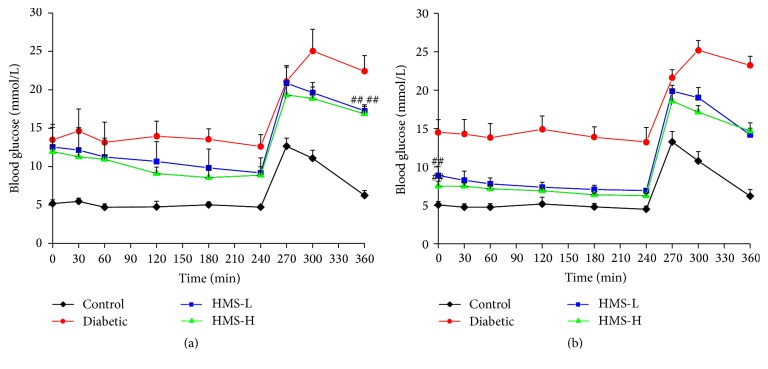
Effects of HMS5552 on oral drug tolerance 1 (a) and 27 days (b) after treatment with HMS5552. HMS-L group, low-dose (10 mg/kg) HMS5552-treated diabetic group; HMS-H group, high-dose (30 mg/kg) HMS5552-treated diabetic group. The data are expressed as the mean ± SD (*n* = 6). ^##^*P* < 0.01, compared with the diabetic group.

The effects of HMS5552 on drug tolerance were studied on days 1 and 27 after i.g. administration. As shown in Figure 3(a), the FPG levels were gradually decreased from 30 min to 240 min in treated rats. Treatment with 10 mg/kg and 30 mg/kg HMS5552 induced decreases of ~27% and 26%, respectively, at 240 min compared with those found prior to HMS5552 administration (all *P* < 0.05), and the 2 h postprandial blood glucose levels were significantly lower in the treated rats than in those belonging to the diabetic group (all *P* < 0.01). After 27 d of treatment, the FPG levels in the HMS-L and HMS-H rats were decreased significantly compared with those detected in the diabetic rats (all *P* < 0.01), presenting reductions of ~22% (*P* < 0.05) and 17%, respectively, at 240 min compared with the baseline level (Figure 3(b)).

### 3.5. Effects of HMS5552 on GK Activity

The *K*_*m*_ value of GK in the liver of the rats belonging to the control group was 7.9 ± 0.32 mmol/L and was significantly lower than that found for the diabetic group (*K*_*m*_ = 9.9 ± 0.55 mmol/L; *P* < 0.01). After one month of treatment, *K*_*m*_ was significantly decreased in both the HMS-L (*K*_*m*_ = 5.9 ± 0.43 mmol/L) and HMS-H (*K*_*m*_ = 5.6 ± 0.27 mmol/L) rats compared with the diabetic rats (all *P* < 0.01).

### 3.6. Effects of HMS5552 on Liver and Pancreas Tissues

#### 3.6.1. Immunohistochemistry Analysis of GK and Insulin

**Figure 4:**
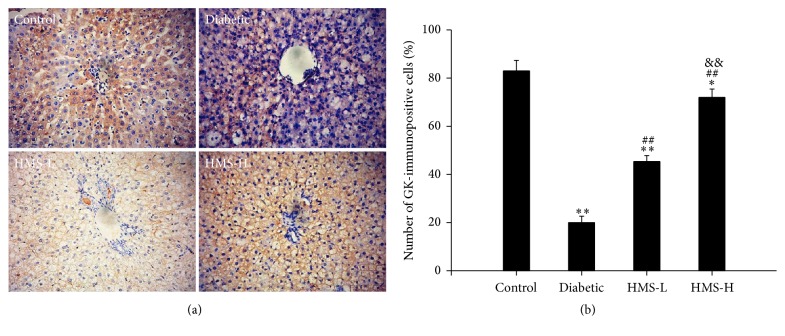
Effects of HMS5552 on GK immunoreactivity in the rat liver. Liver tissue sections stained with anti-GK antibody are shown at 200x magnification (a), and the number of GK-immunopositive cells was analyzed (b). HMS-L group, low-dose (10 mg/kg) HMS5552-treated diabetic group; HMS-H group, high-dose (30 mg/kg) HMS5552-treated diabetic group. The data are expressed as the mean ± SD (*n* = 6). ^*∗*^*P* < 0.05 and ^*∗∗*^*P* < 0.01, compared with the control group; ^##^*P* < 0.01, compared with the diabetic group; ^&&^*P* < 0.01, compared with the HMS-L group.

As shown in Figure 4, GK was mostly expressed in the cytoplasm of the rat hepatocytes. The number of GK-immunopositive cells was significantly decreased in the liver of the diabetic rats compared with the control rats (*P* < 0.01). After treatment with 10 mg/kg and 30 mg/kg HMS5552, the number of GK-immunopositive cells was significantly increased compared with that found in the nontreated diabetic rats (all *P* < 0.01), and the HMS-H group exhibited more GK-immunopositive cells than the HMS-L group (*P* < 0.01).

**Figure 5:**
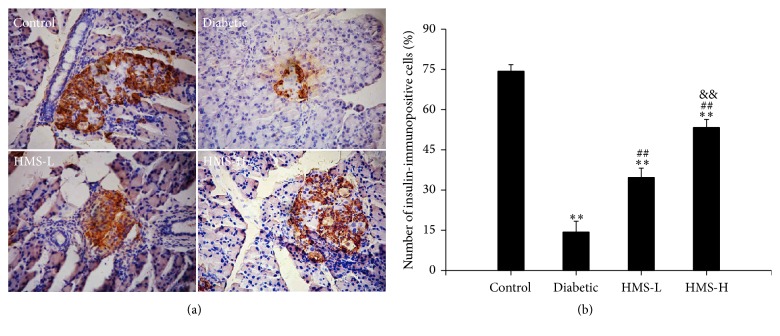
Effects of HMS5552 on insulin immunoreactivity in the rat pancreas. Pancreas tissue sections stained with anti-insulin antibody are shown at 200x magnification (a), and the number of insulin-immunopositive cells in islets was analyzed (b). HMS-L group, low-dose (10 mg/kg) HMS5552-treated diabetic group; HMS-H group, high-dose (30 mg/kg) HMS5552-treated diabetic group. The data are expressed as the mean ± SD (*n* = 6). ^*∗∗*^*P* < 0.01, compared with the control group; ^##^*P* < 0.01, compared with the diabetic group; ^&&^*P* < 0.01, compared with the HMS-L group.

An immunohistochemical analysis of insulin in the pancreas tissues of all groups of rats was performed. The control rats presented strong immunoreactivity to insulin in *β* cells, which occupy most of the islets. The diabetic rats showed a significantly decreased number of insulin-immunopositive cells compared with the control rats (*P* < 0.01). The administration of 10 mg/kg and 30 mg/kg HMS5552 significantly improved the number of insulin-immunopositive cells compared with those found in the diabetic rats (all *P* < 0.01; Figure 5).

#### 3.6.2. Western Blot Analysis of GK

**Figure 6:**
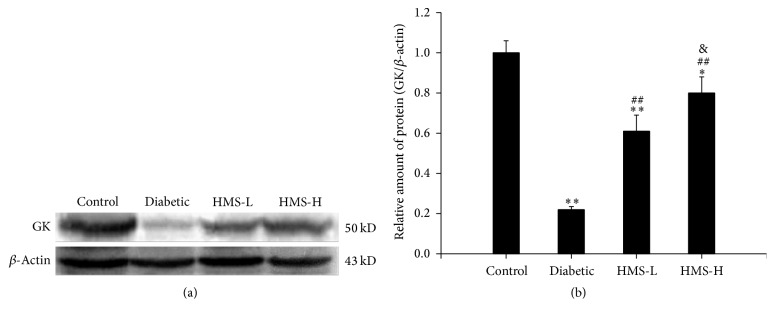
Effects of HMS5552 on the expression of GK protein in the rat liver. The expression of GK was detected by western blot (a), and the intensities of the bands were normalized against that of *β*-actin (b). HMS-L group, low-dose (10 mg/kg) HMS5552-treated diabetic group; HMS-H group, high-dose (30 mg/kg) HMS5552-treated diabetic group. The data are expressed as the mean ± SD (*n* = 6). ^*∗*^*P* < 0.05 and ^*∗∗*^*P* < 0.01, compared with the control group; ^##^*P* < 0.01, compared with the diabetic group; ^&^*P* < 0.05, compared with the HMS-L group.

GK protein expression in the rat liver was analyzed by western blot, and the protein level of GK was significantly decreased in the diabetic group compared with the control group (*P* < 0.01). After the treatment with 10 mg/kg and 30 mg/kg HMS5552, the expression of GK in both groups was significantly higher than that detected in the diabetic group (all *P* < 0.01). Moreover, the expression level of GK protein was significantly higher in the HMS-H group than in the HMS-L group (*P* < 0.05; Figure 6).

#### 3.6.3. Semiquantitative RT-PCR Analysis of GK

**Figure 7:**
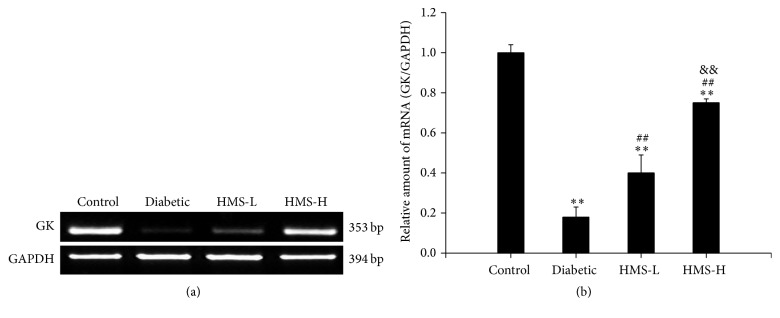
Effects of HMS5552 on the expression of GK mRNA in the rat liver. The expression of GK was detected by semiquantitative RT-PCR (a), and the intensities of the bands were normalized against that of GAPDH (b). HMS-L group, low-dose (10 mg/kg) HMS5552-treated diabetic group; HMS-H group, high-dose (30 mg/kg) HMS5552-treated diabetic group. The data are expressed as the mean ± SD (*n* = 6). ^*∗∗*^*P* < 0.01, compared with the control group; ^##^*P* < 0.01, compared with the diabetic group; ^&&^*P* < 0.01, compared with the HMS-L group.

To assess the effects of HMS5552 on the mRNA expression of GK, a semiquantitative RT-PCR assay was performed. The expression of GK mRNA was significantly decreased in the diabetic rats compared with the control rats (*P* < 0.01), and treatment with 10 mg/kg and 30 mg/kg HMS5552 for one month caused a significant increase in the GK mRNA level compared with the level observed in the diabetic group (all *P* < 0.01). Moreover, the level of GK mRNA in the HMS-H group was significantly higher than that found in the HMS-L group (*P* < 0.01; Figure 7). These results are consistent with the western blot results.

## 4. Discussion

The blood glucose levels are well regulated through a variety of mechanisms, and a number of tissues are involved in this process. It has been demonstrated that GK is a critical enzyme in multiple glucose-sensitive organs: liver, pancreas, brain, and gut [16]. Due to its major role in the regulation of both insulin secretion and hepatic glucose production, GK is an appealing target in the treatment of T2DM. In the current study, we investigated the mechanism of a novel GKA, HMS5552, which mediates the amelioration of glucose metabolism in a rat model of T2DM induced by STZ combined with a HFD. Our data provide evidence supporting the therapeutic effects of HMS5552 on T2DM due to its action in both the liver and pancreas.

A low-dose injection of STZ combined with a HFD is a common method for the establishment of an animal model of T2DM that can imitate the natural history and metabolic characteristics of T2DM patients [17] and is sensitive to pharmaceutical treatment [18]. In the present study, repeated measurements of the FPG levels in the rats of the diabetic group showed levels higher than 8 mmol/L, and the 2 h postprandial blood glucose levels were greater than 22.5 mmol/L. Moreover, the levels of FINC, TC, and TG were significantly increased, whereas the FG levels were significantly decreased, indicating the development of insulin resistance in these animals. Together, these results suggested that a T2DM rat model with insulin resistance was successfully established and was suitable for the subsequent studies.

In addition to the high serum insulin levels, the insulin resistance state of the rats in the diabetic group was confirmed by the severely impaired glucose tolerance [19, 20]. Based on the ΔAUC_0–240 min_ obtained from the OGTT test, the treatment with 10 mg/kg and 30 mg/kg HMS5552 showed obvious improvements in oral glucose tolerance which may be due to insulin secretion from pancreatic *β* cells and increase in the transportation and utilization of glucose. Hyperinsulinemia is another indicator of insulin resistance [21] and contributes to the development of other diseases, such as ischemic heart disease [22] and hypertension [23]. Based on the FINS data, hyperinsulinemia was significantly decreased after one month of treatment with HMS5552, indicating an increase in the insulin-sensitizing activity of HMS5552. These results suggest that HMS5552 exerts its antidiabetic effect by inhibiting insulin resistance and increasing insulin sensitivity.

GK is fundamental to the pathogenesis of T2DM. GK activity is decreased by approximately 50% in T2DM patients compared with controls [24]. Another study demonstrated that GK activity was 4.5-fold lower in hepatocytes from T2DM rats than those from healthy rats [25]. The changes in GK activity in the liver of the T2DM rats observed in this study were closely related to the blood glucose levels. The *K*_*m*_ value of GK in the liver of the diabetic rats was increased by 25% compared with that of the control rats. This result indicates a decreased affinity between GK and glucose in the liver, reflecting a reduced activity of GK, and this finding is consistent with previous results. The HMS-L and HMS-H rats showed decreased *K*_*m*_ values compared with the diabetic rats, which suggests that HMS5552 activates GK. The semiquantitative RT-PCR results confirmed that GK expression was inhibited in T2DM rats and HMS5552 treatment significantly increased GK expression compared with that found in the diabetic group. The above-described outcomes were also validated by immunohistochemistry and western blot analysis.

## 5. Conclusion

In summary, we investigated the action of a novel GKA, HMS5552, in a rat model of T2DM induced by a HFD and the injection of low-dose STZ. The results of our study indicate that HMS5552 can effectively improve GK activity and insulin resistance by targeting both the liver and pancreas. These findings suggest that HMS5552 has potential for the treatment of T2DM, and the compound is currently in Phase II clinical trials.

## Figures and Tables

**Table 1:** Effects of HMS5552 on biochemical parameters.

Group	TC (mmol/L)	TG (mmol/L)	FINS (mU/L)	FG (pg/mL)
Control	4.16 ± 0.92	0.81 ± 0.38	5.33 ± 1.21	71.25 ± 17.85
Diabetic	12.09 ± 3.24^*∗∗*^	3.66 ± 1.60^*∗∗*^	44.42 ± 3.71^*∗∗*^	35.88 ± 4.76^*∗∗*^
HMS-L	11.04 ± 2.75^*∗∗*^	3.43 ± 1.62^*∗∗*^	28.40 ± 4.94^*∗∗*##^	43.38 ± 18.74^*∗∗*^
HMS-H	10.70 ± 3.77^*∗∗*^	3.11 ± 1.14^*∗∗*^	18.74 ± 3.48^*∗∗*##&&^	51.13 ± 4.69^*∗*^

Data are expressed as mean ± SD (*n* = 6); HMS-L group, low-dose (10 mg/kg) HMS5552-treated diabetic group; HMS-H group, high-dose (30 mg/kg) HMS5552-treated diabetic group; TC: total cholesterol; TG: triglyceride; FINS: fasting insulin; FG: fasting glucagon.

^*∗*^ *P* < 0.05, ^*∗∗*^*P* < 0.01, compared with control group.

^##^ *P* < 0.01, compared with diabetic group.

^&&^ *P* < 0.01, compared with HMS-L group.
